# Brain Dynamics Underlying the Nonlinear Threshold for Access to Consciousness

**DOI:** 10.1371/journal.pbio.0050260

**Published:** 2007-09-25

**Authors:** Antoine Del Cul, Sylvain Baillet, Stanislas Dehaene

**Affiliations:** 1 INSERM, Cognitive Neuroimaging Unit, IFR 49, Saclay, France; 2 Atomic Energy Commission (CEA), NeuroSpin Center, Saclay, France; 3 University of Paris XI, Orsay, France; 4 Cognitive Neuroscience and Brain Imaging Laboratory, CNRS UPR640, IFR 49, Paris, France; 5 University Pierre & Marie Curie, Paris, France; 6 Collège de France, Paris, France; University of Oregon, United States of America

## Abstract

When a flashed stimulus is followed by a backward mask, subjects fail to perceive it unless the target-mask interval exceeds a threshold duration of about 50 ms. Models of conscious access postulate that this threshold is associated with the time needed to establish sustained activity in recurrent cortical loops, but the brain areas involved and their timing remain debated. We used high-density recordings of event-related potentials (ERPs) and cortical source reconstruction to assess the time course of human brain activity evoked by masked stimuli and to determine neural events during which brain activity correlates with conscious reports. Target-mask stimulus onset asynchrony (SOA) was varied in small steps, allowing us to ask which ERP events show the characteristic nonlinear dependence with SOA seen in subjective and objective reports. The results separate distinct stages in mask-target interactions, indicating that a considerable amount of subliminal processing can occur early on in the occipito-temporal pathway (<250 ms) and pointing to a late (>270 ms) and highly distributed fronto-parieto-temporal activation as a correlate of conscious reportability.

## Author Summary

Understanding the neural mechanisms that distinguish between conscious and nonconscious processes is a crucial issue in cognitive neuroscience. In this study, we focused on the transition that causes a visual stimulus to cross the threshold to consciousness, i.e., visibility. We used a backward masking paradigm in which the visibility of a briefly presented stimulus (the “target”) is reduced by a second stimulus (the “mask”) presented shortly after this first stimulus. (Human participants report the visibility of the target.) When the delay between target and mask stimuli exceeds a threshold value, the masked stimulus becomes visible. Below this threshold, it remains nonvisible. During the task, we recorded electric brain activity from the scalp and reconstructed the cortical sources corresponding to this activity. Conscious perception of masked stimuli corresponded to activity in a broadly distributed fronto-parieto-temporal network, occurring from about 300 ms after stimulus presentation. We conclude that this late stage, which could be clearly separated from earlier neural events associated with subliminal processing and mask-target interactions, can be regarded as a marker of consciousness.

## Abstract 3

The sequence of neural events associated with the unfolding of conscious awareness is revealed by comparing electrical brain responses to visual stimuli above and below the behavioral threshold for perception.

## Introduction

One of the most obvious and yet unexplained properties of conscious perception is the existence of a threshold for conscious access: when a stimulus is flashed and followed by a backward mask, subjects do not report perceiving it until the target-mask interval exceeds a threshold duration [1,2]. Below-threshold—or “subliminal”—stimuli receive complex perceptual and even semantic processing [3–6], but for an unknown reason, these processes remain inaccessible to consciousness. Understanding the neural mechanisms that distinguish such conscious and nonconscious processes remains a crucial issue in cognitive neuroscience. We used high-density recordings of event-related potentials (ERPs) to ask several questions: (1) What sequence of activations is evoked by subliminal masked stimuli? (2) What additional sequence of brain events leads a stimulus to cross the threshold for conscious reportability? (3) At what time does this access to conscious report occur?

Existing models of conscious access differ markedly with respect to the brain areas involved (posterior versus anterior) and the timing of their activation (early versus late). A first category of model views conscious visual perception as a phenomenon localized to posterior brain areas, and whose contents are determined by the pattern of neuronal activity in early visual and/or occipito-temporal areas [7–13]. According to these proposals, the threshold for conscious perception during masking should be determined solely within the visual cortex, either within a single area or due to short-range recurrent interactions among posterior occipito-temporal regions [10]. As far as timing is concerned, some authors have proposed that conscious perception is already detectable in the ascending, feedforward activation evoked as early as ∼100 ms after stimulus presentation [7]. For others, visual consciousness is not associated with feedforward visual activation, but requires a subsequent period of “localized recurrent processing” [10], still relatively early and confined to posterior occipito-temporal brain systems. Electrophysiological recording in macaque monkeys during masking suggest a peak effect of recurrent interactions in area V1 around 100–140 ms after stimulus presentation [9], and intracranial human recordings suggest that category-specific ventral occipito-temporal cortices are already strongly activated by 150–200 ms, although extension to more anterior regions and reverberation effects can extend for much longer (290–700 ms) [14]. Thus, although predictions concerning timing are less precise, this category of models would predict that subjective reports of conscious perception should correlate with posterior and relatively early brain events.

At odds with this view, a second category of models views conscious access as the formation of a late brain-scale neuronal assembly involving recurrent long-distance interactions among distributed thalamo-cortical regions, particularly the prefrontal cortex and higher cortical association areas [15–28,66]. In a detailed neural network simulation, those areas, when linked by reciprocal top-down and bottom-up connections, exhibit a threshold for “global ignition” [19,20]. When this threshold is exceeded, even a brief external stimulation can simultaneously activate many distant areas and yield a long-lasting pattern of reverberating activity. It is claimed that such a distributed pattern corresponds to a consciously reportable state, because its active contents are broadcast to many specialized processors, including those for verbal or motor report. When an incoming activation fails to exceed the ignition threshold, it can still briefly propagate through the processors but quickly vanishes, because it is not supported by recurrent self-amplifying loops. This state may correspond to a situation of subliminal processing [21].

**Figure 1:**
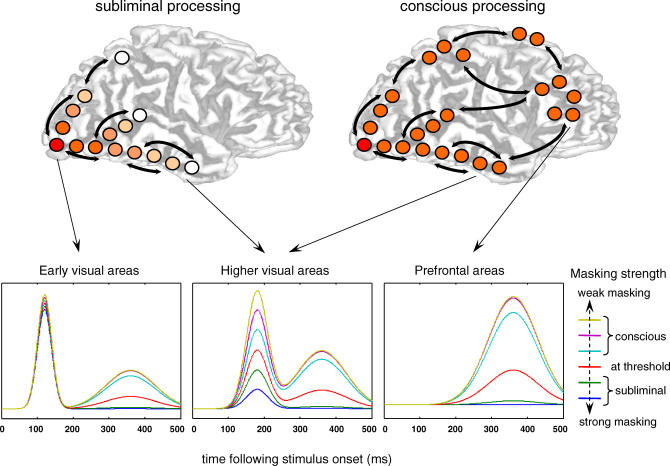
Schematic Predictions Concerning the Brain Activation to Subliminal and Supraliminal Masked Stimuli, as Derived from a Global Neuronal Workspace Model of Consciousness Top, depth of cortical processing: subliminal stimuli (left panel) should evoke a strong activation in extrastriate visual cortex, but their intensity should quickly decrease in higher visual areas; only conscious stimuli (right panel) should trigger a late surge of activation in a global prefronto-parietal network. Bottom, schematic time course of activation as a function of masking strength. Masking is expected to have little effect on early visual activation but to modulate the strength of activation in higher visual areas. Furthermore, there should be a nonlinear effect of masking strength in prefrontal cortex, with a similar late top-down activation peak occurring simultaneously in visual areas [19–21].

Figure 1 illustrates the schematic predictions that may be expected from this “global neuronal workspace” model as a stimulus is made increasingly more visible by lowering its masking strength (for detailed simulations, see [19–21]). First, initial visual activation should be nearly identical for all stimuli, even heavily masked ones, irrespective of whether they are perceived or not. Second, as masking strength decreases, activation should propagate to increasingly deeper levels of processing in occipito-temporal and parietal cortices. Third, when reaching higher cortical areas, particularly the prefrontal cortex, activation should diverge in a nonlinear manner, either quickly building up to a high level (supra-threshold stimulus), or decaying back to baseline (subliminal stimulus). Fourth, the surge of activation to supra-threshold stimuli should occur simultaneously in a global network, including prefrontal, parietal, as well as posterior occipito-temporal regions, thus creating a second, late peak of activation in early visual areas.

**Figure 2:**
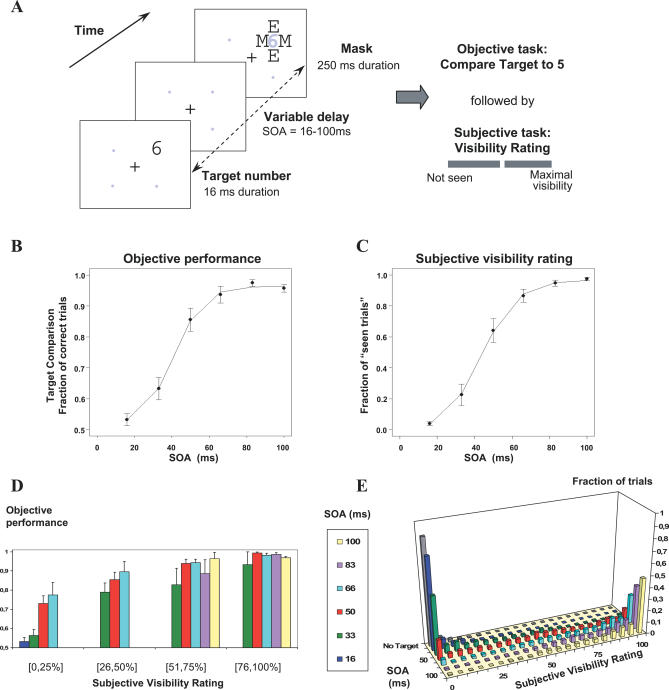
Experimental Protocol and Behavioral Results Collected during ERP Recording (A) On each trial, a target numeral (16 ms) was presented at one of four possible location, and followed by a letter mask (250 ms). Gray dots and numerals are shown for spatial reference only, and were not actually presented. Six conditions of target-mask SOA=16, 33, 50, 66, 83, or 100 ms and one mask-only condition (without target) were randomly intermixed across trials. Following each such stimulus, subjects performed two consecutive tasks: (1) forced-choice comparison of the target numeral with the numeral 5 and (2) evaluation of subjective target visibility using a continuous scale labelled “not seen” at left and “maximal visibility” at right. (B and C) Objective and subjective measures of conscious perception. (B) Percentage of correct responses in numerical comparison as a function of SOA. (C) Proportion of trials subjectively rated as “seen” as a function of SOA. In both graphs, the sigmoid curve fitting the data is represented as a continuous line. Error bars represent the standard error. (D) Objective performance at different levels of subjective visibility and SOA; only cells with at least five measures per subject are included. (E) Distribution of subjective visibility ratings yielding the mean data shown in (C).

To test these predictions, we collected high-density recordings of ERPs during a backward masking paradigm [2] in which a single brief (16 ms) parafoveal target digit was followed by a mask consisting of letters surrounding the target location (see Figure 2). We varied quasi-continuously the target-mask stimulus onset asynchrony (SOA) so that, with increasing SOA, the same target stimulus crossed the threshold from subliminal processing to conscious perception. We documented this transition behaviorally using objective (forced-choice comparison of the digit to five) and subjective (continuous scale of visibility) measures of conscious access. Both the proportion of “seen trials” and the objective performance increased nonlinearly as a function of SOA, thus tracing a characteristic sigmoidal curve with a well-defined threshold (Figure 2).

Recordings of ERPs evoked by these stimuli then allowed us to examine how brain activity changed around this transition point. By subtracting out the brain activity evoked by the mask only, we isolated the entire sequence of target-evoked ERPs, thus allowing us to visualize both early visual activation and later brain events. To determine which of these components were associated with conscious-level processing, we used two independent criteria. First, we searched for ERPs whose profile of amplitude as a function of SOA traced a nonlinear curve parallel to the proportion of “seen” trials. In other words, we used this nonlinear profile of subjective visibility ratings as a “signature” of conscious processing in the brain (as done in functional MRI (fMRI) by Haynes et al. [29] for metacontrast masking). As a second criterion, we also examined, at a fixed intermediate SOA value, the difference between trials reported as “seen” and trials reported as “not seen.” The central issue was whether those two criteria (nonlinear signature and difference between seen and not-seen reports) pointed to an early activation localized to occipito-temporal areas or to a late global ignition in prefrontal and parietal areas.

## Results

### Behavioral Results

We first evaluated the subjects' objective and subjective perception of masked digits (Figure 2B–2E). Figure 2E illustrates how subjective visibility ratings neatly clustered into two well-defined “seen” and “not-seen” states. When the target was absent, subjects hardly ever gave subjective ratings exceeding 25% of the scale (0.3% of trials). Conversely, at the longest SOA where the target was highly visible, the ratings almost always exceeded 25% of the scale (97.4% of trials). At intermediate SOAs, the distribution of cursor positions reached a minimum around 25%, separating the data into two sets, one peaking at visibility zero (“not-seen” trials), and the other at maximal visibility (“seen” trials), replicating our earlier observations [2,30,31]. Thus, the value of 25% visibility was used as a cut-off between seen and not-seen trials.

We then calculated for each SOA and each subject the percentage of seen trials (Figure 2C). The percentage of seen trials increased significantly as a function of SOA (F(5,55) = 154.17, *p* < 0.001). The increase was nonlinear and showed a quick transition between 33 and 66 ms from mostly not-seen to mostly seen responses. Indeed, there was a significant difference (*p* < 0.01) between the rate of increase of subjective visibility ratings in the SOA interval from 33 to 66 ms and that in the 16–33 ms and 66–83 ms intervals, indicating a significant nonlinearity. For each subject, the curve relating the percentage of seen trials to SOA was well fitted by a sigmoid. The subjective visibility threshold was defined as the SOA where the sigmoid reached its inflexion point (for mathematical details, see Materials and Methods and [2]). The mean subjective threshold was 43.9 ms (standard deviation [SD]= 10.5 ms).

Similarly, in the objective task, performance in comparing the target with the numeral 5 increased with SOA (F(5,50) = 82.02, *p* < 0.001; see Figure 2B). This performance was at chance level only for the shortest SOA of 16 ms. Again, the rate of increase was larger in the SOA interval from 33 to 66 ms than in the 16–33 ms and 66–83 ms intervals (*p* < 0.05). A sigmoid provided a good fit and allowed us to define an objective visibility threshold for each subject (mean = 40.8 ms; SD =. 12.4 ms).

As in our previous work [2], a strong linear correlation was found between objective and subjective thresholds (*r* ^2^ = 0.747, slope = 1.03), once a single outlier subject was excluded. This indicated that subjective and objective measures followed a similar nonlinear transition with a single threshold parameter which varied across subjects. To further study the relation between subjective and objective measures of conscious access, we subdivided the subjective visibility scale into four bins according to the position of the cursor (1st, 2nd, 3rd, and 4th quarters of the scale, with the 1st quarter corresponding to the above “not-seen” category). We then computed the objective performance for the different SOA within each of these four subjective bins (Figure 2D). There was a good cross-validation of the objective and subjective measures inasmuch as objective performance was always way above chance at all SOAs whenever trials were classified as “seen” (bins 2, 3, and 4). Conversely, in bin 1 corresponding to not-seen trials, objective performance was at chance level for SOAs 16 and 33 ms (*p* = 0.11 and *p* = 0.059, respectively), but above chance for longer SOAs 50 and 66 ms (*p* < 0.001). Those results indicated that on some trials, subjects could compare accurately the target to the numeral 5 even if they reported that they could not see it. Such above-chance performance in the absence of conscious reportability is reminiscent of “blindsight” and is indicative of subliminal processing, as previously reported in subliminal priming tasks [5,32,33].

In summary, both objective and subjective data followed a sigmoidal curve as a function of SOA, with a similar nonlinear threshold (defined as the inflection point of the sigmoid). We take this parallelism between objective and subjective measures of conscious access as an indication that a major transition in processing occurs around SOA = 50 ms, and this transition affects a broad range of cognitive processes, including those leading to introspective reports as well as those leading to simple numerical decisions. We are mostly concerned with the neural correlates of this major transition, which is captured by both objective and subjective measures. Note that our definition of the conscious threshold departs from the classical psychological definition, which is based on the SOA at which objective performance first departs from chance. Indeed, a partial dissociation was found at short SOAs, where we observed a proportion of subjectively not-seen trials accompanied by above-chance objective performance. This dissociation is compatible with the notion of a distinct “objective threshold” within the zone associated with an absence of subjective perception [34]. Unfortunately, however, there were too few trials in this “gray zone” between objective and subjective definitions of conscious perception to address the issue of its underlying brain mechanisms. We focused our analysis on the correlates of the subjective threshold and, more precisely, on the issue of which ERP components exhibited a nonlinear curve parallel to the subjective reports. As noted above, behavioral visibility ratings increased suddenly from SOA = 33 ms to SOA = 66 ms, whereas they varied little either from 16–33 ms or from 66–83 ms. This specific shape was used as a litmus test to identify which ERP components correlated with conscious access. As a second criterion, we also examined which ERP components distinguished seen and not-seen subjective reports at the threshold SOA of 50 ms.

### ERPs

#### Separation of activation evoked by the mask and by the target.

**Figure 3:**
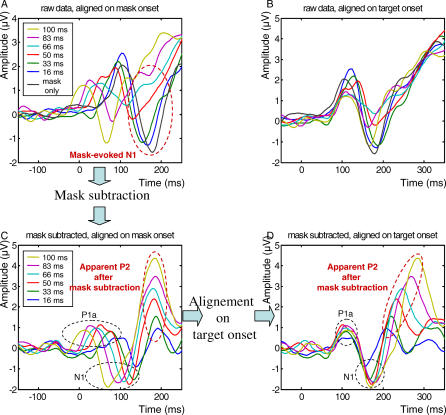
Mask Subtraction Method The mask subtraction method is illustrated here for ERP activity recorded in parieto-temporal electrodes controlateral to the hemifield of stimulus presentation (averaged across right and left stimulus presentation). To separate activity evoked by target from activity evoked by the mask, the following procedure was used: (A) Alignment of ERPs on mask onset; (C) Subtraction of ERPs evoked by the mask-only condition from each of the others six target + mask conditions; (D) Realignment of these subtracted ERPs on target onset. For comparison, (B) shows the nonsubtracted ERPs aligned on target onset. The subtraction procedure allowed to recover two target-evoked components (P1a and N1) and one later mask-evoked component (P2).

One difficulty with the present paradigm is that both the target and the mask evoke ERPs of their own, which become superimposed at different times as a function of SOA (Figure 3). To subtract out the activity evoked by the mask, we first aligned the ERPs on the mask onset; then we subtracted the ERP evoked by the mask alone from each of the other target + mask conditions; and finally, we realigned those subtracted data on target onset. Figure 3 illustrates how this procedure recovers the N1 component evoked by the target. After mask subtraction and realignment on target onset, the N1 becomes clearly visible, properly aligned for all SOAs, and with a similar latency relative to target onset. Time-locking to the target was assessed statistically by a linear regression, which examined whether the peak latency varied with SOA when measured either relative to target onset or to mask onset. When measured relative to target onset, the N1 latency did not vary with SOA, whereas when measured relative to mask onset, it varied linearly in inverse relation with SOA (see statistics in Table 1), thus indicating time-locking with the target.

**Table 1:**
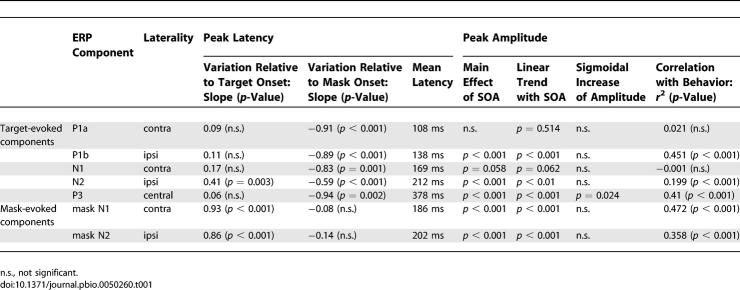
Summary of the Results of Statistical Tests Performed for Peak Amplitude and Latency of the Different Target-Evoked and Mask-Evoked Waveforms.

**Table 1:**
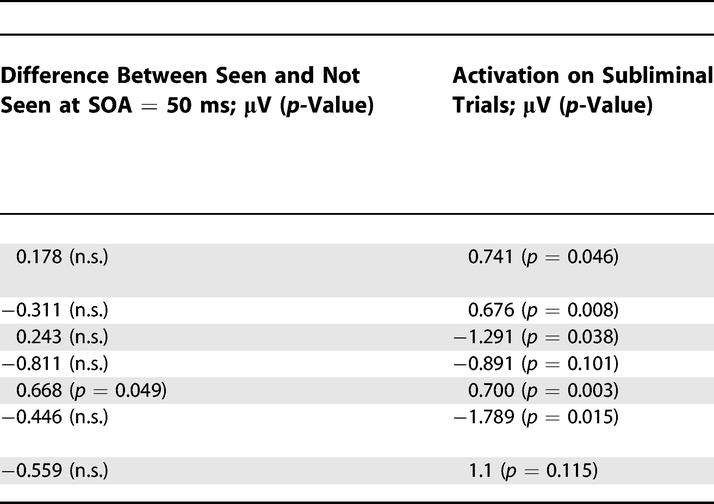
Extended.

**Figure 4:**
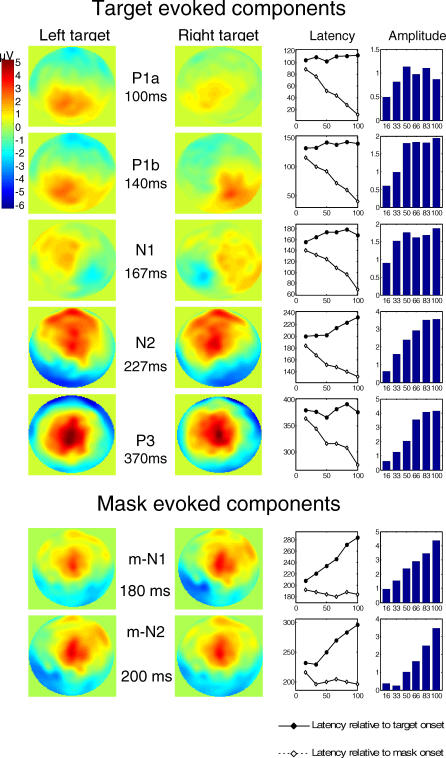
Sequence of ERP Components Evoked by the Target and Mask Five ERP components were found to be elicited by the target, and two other components were found to be evoked by the mask. These two last components appeared as positive waveforms since they result from the subtraction of mask-only activity from the mask-evoked N1 and N2 activity for the different target + mask conditions. In the mask-subtracted data, these components appear to increase with SOA, which in reality means that the mask-induced activation (mask-evoked N1 and N2) decreases with SOA. For each component, its peak latency (in milliseconds) is represented as a function of SOA measured relative to target (continuous line) or to mask onset (dashed line). The histograms at right show peak amplitude (in microvolts). Their topography in response to left and right hemifield stimulation is illustrated by voltage maps of the scalp surface, for the target + mask after mask subtraction, SOA=100 ms (first five target-evoked components) and for the mask-only condition (for mask-evoked N1 and N2 components).

**Figure 5:**
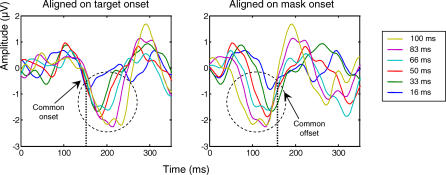
Interruption of Target-Evoked Activity by Mask Presentation Mask-subtracted ERPs from occipito-temporal electrodes, averaged across right and left hemifields of stimulus presentation, are represented aligned respectively on target onset (left panel) and mask onset (right panel). The N2 component shows a common onset for all SOA conditions when ERPs are aligned on target onset, and a common offset when ERPs are aligned on mask onset. Thus, the N2 may reflect a process that starts with target onset and is interrupted by the mask.

Application of this procedure led to the identification of five main ERPs evoked by the target (Figure 4). The scalp topography of the first four (P1a, P1b, N1,and N2) varied according to the stimulated hemifield, whereas the latest (P3) did not. Regressions on peak latencies (Table 1) confirmed that most of these components (P1a, P1b, N1, and P3) were solely time-locked to target presentation. The N2, however, had singular properties, because its peak latency varied with SOA both relative to mask onset and to target onset. The time course of this waveform (Figure 5) suggested that, across the different SOAs, its onset was time-locked to the target, whereas its peak and offset were approximately time-locked to the mask. This was confirmed by linear regressions. The N2 onset was invariant relative to target onset (regression with SOA, slope = 0.08, not significant), but varied with SOA relative to mask onset (slope = −0.92, *p* = 0.001). Conversely, the N2 offset increased relative to target onset (slope = 0.55, *p* = 0.006), although it also shifted slightly relative to mask onset (slope = −0.45, p = 0.012). Thus, this N2 appears to index a visual process that starts at a fixed interval after the target onset, and the N2 is interrupted at a fixed interval after the mask onset.

Surprisingly, even after subtraction of the mask-only ERP, two other ERP components remained time-locked to the mask onset (Figure 4). A simple explanation is that these components were evoked by the mask but their amplitude varied with the target-mask SOA, hence preventing their removal by simple subtraction of a fixed ERP evoked on mask-only trials. Indeed, the latency and topography of these components suggest that, before subtraction of the mask-only ERP, they corresponded to the N1 and N2 evoked by the mask (here after called m-N1 and m-N2). Both components were of high amplitude in the mask-only condition and at short SOAs in the target + mask conditions, but progressively vanished as the SOA increased (Figure 3AA). As a consequence, the subtraction procedure (computing the target + mask ERP minus the mask-only ERP) created artificial positive peaks whose amplitude seemed to increase with SOA (Figure 3C and 3D). By their latency and amplitude variations, however, we could clearly assign these components to mask-evoked processes that progressively lost the competition with the target and therefore diminished in amplitude as the SOA increased.

In summary, two types of ERP components were identified by our mask subtraction procedure. Five components (P1a, P1b, N1, N2, and P3) appeared to be preferentially evoked by the target and to increase in amplitude when SOA increases. Two other components seemed to correspond to an activation evoked by the mask (mask-evoked N1 and N2) and to decrease in amplitude when SOA increases.

#### Correlation of ERP components with subjective reports.

We examined each of these components to see whether their amplitude varied with SOA with a profile similar to subjective visibility reports (Table 1). Two target-evoked components, the P1a and the N1, showed no significant amplitude variations. These components are essentially unaffected by masking (although a nonsignificant trend toward reduced amplitude at SOA = 16 ms can be seen), confirming earlier reports of preservation of these components independently of the subject's conscious report [30,35–37]. The other components (P1b, N2, m-N1, m-N2, and P3) increased significantly in amplitude with SOA. As a consequence, they all correlated partially with subjective reports, which also increased with SOA. Nevertheless, finer analysis showed that only the P3 component exhibited the characteristic nonlinear signature seen in subjective reports (Table 1). Other components showed a fast increase in amplitude as SOA increased from 16 to 50 ms followed by a shallower asymptote. These observations were confirmed statistically by testing whether the amplitude increased in a sigmoidal fashion, i.e., whether the increase in amplitude between SOAs of 33 and 66 ms (surrounding the subjective consciousness threshold) was greater than the combined increases between 16 and 33 ms and between 66 and 83 ms, as previously found with subjective and objective behavioral measures. This nonlinear sigmoidal contrast was only significant for the P3, not for any of the earlier components (see Table 1). To explicitly test the hypothesis that the nonlinearity emerged suddenly around the time of the P3, and was therefore stronger for the P3 than for the preceding N2, we computed a *t*-test across subjects on the difference between the non-linear sigmoidal contrasts for P3 and for N2 (after flipping the sign of the N2 voltages, because the N2 and P3 are of opposite polarities). A significant difference was observed (*t*(11 degrees of freedom [df]) = 2.70, *p* = 0.021). The difference remained significant even after the contrasts were normalized to compensate for the fact that the P3 was, on average, slightly larger than the N2 (1.67 versus 1.30 μV; *t*(11 df) = 2.34, *p* = 0.039). Thus, this analysis confirmed that a significant change occurred between the N2 and the P3 time windows, with a pronounced linearity present in the latter but not the former. Although there was a clear impact of SOA on prior brain activation, with a progressive increase in most target-evoked components (except for P1a and N1) and a corresponding decrease in mask-evoked components as SOA increased, it was not until the P3 that a clear sigmoidal curve characteristic of subjective report was seen.

**Figure 6:**
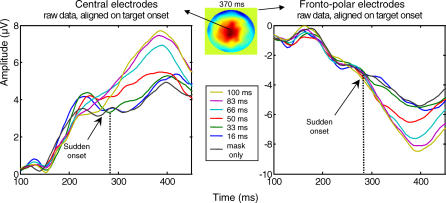
Sudden Onset of a Nonlinear Variation of Evoked Activity with SOA The figure shows the mean amplitude of nonsubtracted ERPs aligned on target onset, measured on central (left panel) and fronto-polar electrodes (right panel). The voltage map shows the topography at the peak of the P3 waveform (latency 370 ms), in the SOA = 100 ms condition. In both graphs, ERPs show a nonlinear increase in amplitude with SOA, with a sudden onset around 270–300 ms.

To clarify further the exact onset of this sigmoidal effect, Figure 6 shows the evolution of ERPs on both central and fronto-polar electrodes. Although voltage departed from zero as early as about 150 ms following target onset, a sharp divergence between the SOA conditions, with a sigmoid shape, was only seen at about 270–300 ms after target onset.

#### Effect of subjective visibility at threshold.

**Figure 7:**
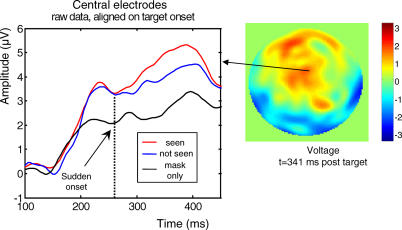
Difference in Activity Evoked by Conscious and Nonconscious Masked Stimuli Left panel, mean amplitude of mask-subtracted ERPs measured on central electrodes is represented respectively for seen and not-seen trials, for SOA = 50 ms, for a subset of nine subjects who had enough measures in both conditions. Identical activity is initially observed in both conditions, but a divergence is seen starting around 270 ms, with seen trials generating an increased positivity. This difference is similar to the nonlinear divergence as a function of SOA observed on the same electrodes (see Figure 5). Note that not-seen trials evoked greater activity than mask-only trials, indicating that late subliminal activity was induced by the unseen targets. The right panel shows the topography of the difference between seen and not-seen trials at SOA = 50 ms.

As a second independent criterion for a cerebral correlate of conscious access, we asked which ERP components varied with subjective reports when the visual stimulus was fixed. To this aim, we examined the trials with an SOA of 50 ms, close to the conscious threshold, and sorted them as a function of whether the subject reported seeing the target or not (three subjects had to be excluded from this analysis because they did not have enough observations in one of these two categories). The only significant difference between seen and not-seen trials was again found on the target-evoked P3 component (Table 1). As shown in Figure 7, on central electrodes, seen and not-seen trials initially generated identical activity, but a divergence was seen starting around 300 ms, with seen trials generating an increased positivity. This difference was quite similar to the nonlinear divergence observed on the same electrodes as a function of SOA (compare Figures 6 and 7). Thus, both criteria clearly point to the P3 as an important correlate of conscious perception.

Importantly, at SOA= 50 ms we also observed that ERPs were larger on not-seen trials compared to mask-only trials, even in the late part of the epoch (see Figure 7). To quantify this subliminal activation effect, we used a *t*-test to compare, for each ERP component, the amplitudes of the ERPs evoked on target-present not-seen trials at SOA = 50 ms and on mask-only trials. As shown in Table 1, almost all components showed a significant subliminal target-presence effect, the sole exception being the N2 (*p* = 0.10). Thus, not-seen targets at SOA = 50 ms elicited an entire sequence of ERP component including a partial P3-like activity. The effect of conscious visibility merely manifested itself as a sudden amplification of the late part of this activation stream.

#### Source analysis of cortical dynamics.

**Figure 8:**
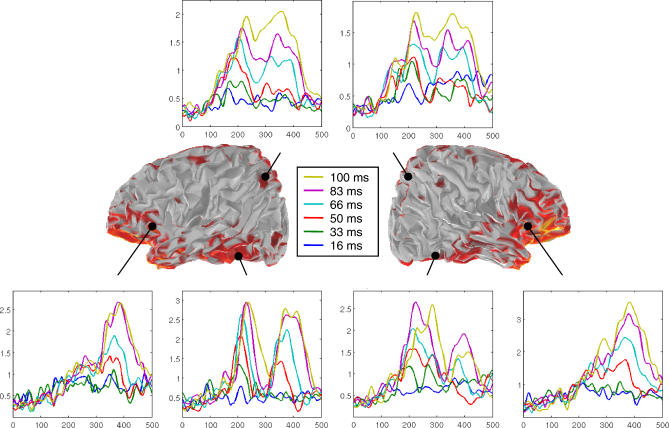
Sequence of Cortical Activity Evoked by the Masked Targets Left and right views of the partially inflated hemispheres show the reconstructed cortical sources at the peak of the P3 (370 ms) in the condition of maximal visibility (SOA = 100 ms). Colors indicate the activation of reconstructed cortical sources, expressed in current density units (A.m), thresholded at 50% of the maximum (yellow = 10^−−7^ A.m). Insets show the profiles of mask-subtracted activity (average of absolute current density) in six bilateral regions of interest (right and left posterior parietal, posterior ventral temporal, and inferior frontal areas), separately for the six conditions of SOA. Two phases of cortical activation can be distinguished. In a first phase, prior to about 300 ms, activation progresses from the occipital pole toward both parietal and ventral temporal sites, and its amplitude increases roughly linearly with target-mask SOA. In a second phase, after 300 ms, there is a sudden onset of high-amplitude activity, with a sigmoidal profile, particularly in ventral prefrontal cortex, accompanied by a concomitant reactivation of all previous posterior sites.

To shed some light on the cerebral generators of this scalp-recorded activity, we modeled the scalp-evoked ERPs using a large number of distributed dipoles spread throughout the cortical surface. Although the spatial precision of this method is limited, it can reconstruct the approximate distribution of activity on the inflated cortical surface and estimate the temporal profile of activation in various regions of interest. Figure 8 shows these average profiles as a function of SOA for right and left hemispheres. Figure S1 shows activation separately for controlateral and ipsilateral stimulation in a larger number of regions.

A precise sequence of cortical events was observed. Activation began around 85 ms in the occipital cortex controlateral to the stimulus, corresponding to the P1a. It was present at all SOAs (Figure S1A1). Starting around 115 ms, activation spread to the ipsilateral occipital cortex and bilaterally to the posterior parietal cortex (Figure S1A2–S1B2). This activation, corresponding to the scalp P1b, was seen only for SOA > 33 ms. After 150 ms, activation corresponding to the scalp N1 was seen as an increase in contralateral occipital and parietal activity, with an additional anterior progression within contralateral ventral temporal cortex (Figure S1A1, S1B1, S1C1, and S1D1). Around 180 ms, activation spread to homolog ipsilateral regions and became bilateral (corresponding to the scalp N2; Figure S1A–S1D). Furthermore, activation now extended anteriorily toward the bilateral temporal, parietal and frontal poles (Figure S1B, S1E, S1F, and S1G). In most regions, these activations increased monotonically with SOA. A notable exception was the contralateral mid-ventral temporal cortex, where initial activation intensity was the same for all SOAs > 16 ms (corresponding to the scalp N1; Figure S1D1). This region thus activated quite strongly to subliminal symbolic stimuli, as previously reported with fMRI [18].

A cortical correlate of the interruption of target-induced activity by the mask (corresponding to the scalp N2) was seen in the posterior ventral temporal cortex. Here the initial wave of activity was brutally interrupted about 180 ms after the mask appeared. Thus, its duration appeared to be proportional to SOA (Figure S1C; compare with the scalp N2 shown in Figure 5). Interestingly, in more anterior ventral temporal regions, activity did not show a similar collapse but tended to show only a small temporary dip or to remain sustained and unaffected, particularly for long SOAs (Figure S1D and S1E). More generally, as one progressed forward in the occipito-temporal pathway, the amplitude of target-induced activation became increasingly differentiated as a function of SOA, with an ordering increasingly similar to subjective reports.

From 200 to 300 ms, we also observed activations linked to the mask, first in controlateral occipital, posterior ventral temporal, and parietal cortices (corresponding to the scalp m-N1; Figure S1A1, S1B1, and S1C1), then ipsilateral to stimulus presentation in occipital and ventral temporal cortex (corresponding to the scalp m-N2; Figure S1A2). This activation propagated anteriorily in bilateral ventral temporal cortices (Figure S1D and S1E).

Finally, starting around 300 ms, target-evoked activation associated with the scalp P3 quickly expanded to a broad fronto-parieto-temporal network. Crucially, its activation occurred only for SOAs associated with conscious reports (Figures 8 and S1). Ventral frontal regions showed a particularly sudden burst of activation whose intensity traced a sigmoidal curve comparable to subjective reports. However, this effect was not restricted to the ventral frontal cortex, but it was very global and seen with a smaller amplitude in most frontal, parietal, and temporal regions of interest. It was broadly distributed to bilateral cortical areas, regardless of the hemifield of stimulus presentation, in agreement with the hypothesis that a global cortical “broadcasting” underlies conscious reportability [15–17,19,20]. In posterior regions, this late sigmoidal peak of activation was well differentiated from the initial stimulus-induced peak (see Figure 8), thus resulting in a bimodal activation profile. This profile fits with previous empirical observations and theoretical expectations that two distinct feedforward and feedback stages should successively occur in visual areas, only the second of which correlates with subjective reports [8,9,19,20].

In summary, two phases of cortical activation could be distinguished. In a first phase, before about 300 ms, activation progressed from the occipital pole toward both parietal and ventral temporal sites, with a shift from initial contralaterality to late bilaterality, and with a progressive attenuation as the target-mask SOA was shorter. In a second phase, only seen for SOAs associated with conscious reportability, there was a sudden onset of high-amplitude activity in the ventral prefrontal cortex, with a concomitant reactivation of all previous posterior sites. Some areas only showed the second phase of activation (e.g., bilateral inferior prefrontal regions), others showed sustained activity bridging across the two phases (e.g., bilateral parietal regions), and yet others a bimodal pattern with a clear gap between the two phases (e.g., inferior occipito-temporal) (Figure 8).

We also examined the distributed cortical sources corresponding to our second criterion for a correlate of conscious access, namely the difference between seen and not-seen trials at SOA = 50 ms. As shown in Figure S2, at the peak of this effect, 340 ms after stimulus presentation, a global fronto-parieto-temporal network was again observed. However, compared with the above network isolated by its sigmoidal response profile, the seen versus not-seen network also included a broad activation increase along the ventral occipito-temporal pathway. The time course of activation in these regions again showed a first wave (<270 ms) identical for seen and not-seen trials, followed by a surge of activation solely in the seen condition. The latter effect started about 270 ms after stimulus onset and lasted until about 400 ms, thus precisely coinciding with the time window where the nonlinear SOA effect was found (Figures 6 and 8; see also Figure S2).

## Discussion

### Summary of Main Results

Our procedure isolated the brain activity evoked by a masked visual stimulus and examined how it was affected by backward masking at variable target-mask SOAs. Although several ERP components were affected by backward masking, two criteria suggested that the P3 component was most tightly associated with subjective perception: (1) its amplitude as a function of SOA exhibited the same sigmoidal shape as the fraction of seen trials; and (2) it showed a significant difference between seen and not-seen trials at a fixed SOA. Source analysis suggested that the underlying cerebral mechanism was a sudden activation of a distributed bilateral fronto-parieto-temporal network starting about 270 ms after stimulus onset. In the preceding period (140–270 ms), the results revealed the progressive build-up, in posterior occipito-temporal and parietal areas, of a nonlinear divergence of activation as a function of SOA, which points to a dynamic nonlinear amplification as the neural correlate of the masking threshold.

### Brain Activity below the Conscious Access Threshold

A first important outcome of our experiment is to characterize further the fate of subliminal masked stimuli. Our results confirm that nonreportable visual stimuli can propagate through a series of cortical processing stages [1,3–6]. As suggested by several previous behavioral experiments [38,39], they indicate that the depth of processing depends on the target-mask interval (SOA). At the shortest SOA (16 ms), there was a strong reduction in all ERP components (though not reaching significance for the P1a). This observation suggests that masking at such short target-mask interval may already partially occur at a peripheral or subcortical level, thus leaving little target-induced activation except in early occipital cortex. As soon as SOA reached 33 ms or more, however, we observed strong subliminal activation in the contralateral occipito-temporal pathway (particularly the mid-ventral temporal cortex, where the activation corresponding to scalp N1 had the same intensity for all SOAs > 16 ms), with a small spread to ipsilateral temporal and bilateral parietal cortices.

It could be argued that at SOA = 33 ms, the averaged ERPs were contaminated by a non-negligible proportion of seen trials, because the sigmoidal curves for objective and subjective reports had already taken off at this SOA value. Additional analyses, which are presented in Figure S3, however, demonstrated that the results were essentially unchanged when excluding the minority of seen trials and restricting the analysis to not-seen trials at SOA = 33 ms. Thus, early visual processing indexed by the P1a and N1 is essentially unaffected even on trials subjectively rated as invisible.

This conclusion is compatible with previous single-cell recordings during backward masking paradigms, which revealed preserved selective firing in occipital [9,40,41] and inferior temporal cortices in response to heavily masked stimuli [42,43]. They also corroborate previous human ERP data, demonstrating preservation of P1 and N1 waveforms during backward masking and attentional blink paradigms [30,35–37], Similarly, recent fMRI studies of backward masking paradigm have shown that early visual activity need not be reduced under conditions of stimulus invisibility [29,44]. Even under conditions of strong metacontrast masking, visual activation is maintained in areas V1 and V2 [12] and contains sufficient information to allow partial stimulus decoding [6].

By sorting trials using subjective reports of “seen” versus “not seen,” we also examined the cerebral processing of stimuli presented for an even longer target-mask interval (50 ms) and yet still reported as being invisible. Those result (Figure 7) indicated that at these lags, an even longer-lasting subliminal activation, extending beyond early visual processing, can be induced. Indeed, all ERP components, including the P3, showed a significant subliminal activation relative to target-absent trials. Nevertheless, although activation amplitudes were initially identical between seen and not seen stimuli before 270 ms, this activation then decreased in amplitude relative to seen trials (Figure 7), and cortical source models indicated that it dropped toward zero within about 500 ms (Figure S2, green curve, lower panel).

We thus conclude that a brief subliminal stimulus can produce a transient activation in many areas, even those associated with the scalp P3 waveform, but that this activation is small and brief. This conclusion fits with several previous observations of anterior brain activation under subliminal conditions, including frontal eye field activation by masked stimuli in monkeys [45], anterior negativity evoked by undetected errors [46], and frontal and cingulate P300 responses evoked by subthreshold visual oddballs [47,48]. In these reports as in the present data, the subliminal activation was always strongly reduced compared to the supraliminal case. As discussed further below, a minimal threshold level of activation seems to be needed to cause a global and sustained ignition associated with conscious perception.

### Target-Mask Interactions

A second contribution of the present results is to visualize some of the cortical interactions by which a retrograde mask can interfere with a preceding visual target and prevent its conscious perception. Masking must be caused by an interference of the mask on the brain activity induced by the target, but its locus remains debated [1,9,42,43,49]. Our source localization data provide a fine characterization of the interactions between target and mask. We first identified an early local competition reflected in the target- and mask-evoked N1 components: as SOA increases, the target N1 increases in amplitude whereas the mask N1 decreases. Source analysis indicates that this antagonistic relation occurs in contralateral occipital, posterior parietal, and posterior ventral temporal cortices. This result is compatible with the hypothesis of a competition between the early visual events evoked respectively by the target and mask, as postulated in many models of masking [1,49].

At a later stage, a similar antagonistic relation occurs at the level of the N2 waveforms evoked by the target and the mask, and this is associated with a distributed bilateral activation of ventral occipito-temporal and posterior parietal cortex. We found that the target-evoked N2 starts at a fixed latency relative to target presentation and continues until it is interrupted by mask-induced activity. Interestingly, neurophysiological recordings in macaque inferior temporal cortex (IT) during a picture masking paradigm have identified single neurons with a similar response profile [42]. Exactly as in the present N2 component, these neurons start firing selectively at a fixed latency after picture onset, but they suddenly cease firing at a fixed latency after mask onset. The N2 might thus represent an ERP signature of this neuronal activation of IT cortex.

Both findings indicate that in spite of their brief initial presentation (16 ms), visual stimuli can evoke durable activation in a posterior network of areas. The mask presentation has the effect of terminating this activation. This effect of the mask is compatible with interruption models of masking, according to which the presentation of the mask erases the cortical representation of the target by suddenly interrupting target-induced ongoing activity in a visual buffer [1,49,50].

The N2 may thus index a stage of cortical processing at which the representation of a brief visual target progressively gains in strength within a distributed set of posterior occipital, temporal, and parietal areas as masking strength decreases. Yet the N2 still fails to meet our two criteria for a genuine correlate of conscious access: (1) its activation increases linearly rather than sigmoidally with SOA, and (2) at at fixed SOA of 50 ms, it does not differ for seen versus not-seen trials. Thus, we conclude that this waveform does not yet constitute a correlate of conscious access stricto sensu. The fact that it shows a relatively high level of activation at short SOAs of 33 and 50 ms (Figure 4) indicates that its activation strength is not always predictive of conscious access.

### Cerebral Correlates of Conscious Access

Our two criteria for conscious access were only met at a later time period, ranging from 270 ms to about 400 ms, and were associated with a broad fronto-parieto-temporal network. During this late time period, which coincides with the scalp-evoked P3 waveform, (1) the neural activity evoked by masked stimuli varies nonlinearly with SOA in parallel to the sigmoidal behavioral curve and (2) its amplitude differs between seen and not-seen trials.

This conclusion is nicely corroborated by a recent study of metacontrast masking using magnetoencephalography [51]. Van Aalderen et al. compared two masking procedures, one of which lead to an absence of subjective conscious perception (effective mask) whereas the other did not (pseudomask). In both cases, the target-mask SOA was varied in small steps, as in the present study. While several MEG components varied with SOA, it was not until a late time window (290–390 ms) that a significant correlate of conscious access was observed, in the form of a U-shaped activation curve only found in the effective mask condition and tightly paralleling subjective reports.

The hypothesis of a late correlate of conscious access also meshes well with a previous experiment from our laboratory using a different paradigm for rendering stimuli invisible, the attentional blink [30]. In this study, Sergent et al. contrasted the neural activity evoked by words which were either consciously perceived or attentionally “blinked” by a concurrent task. When comparing seen and not-seen trials, a critical transition in neural activity was also found from 270 ms after stimulus presentation. As in the present study, the transition was also preceded by an intermediate period of divergence between 170 and 300 ms, and was followed by an all-or-none spreading of activity to a distributed fronto-parieto-temporal network similar to the present one.

Sergent et al. only contrasted ERPs in two quite distinct states: during the attentional blink (T1-T2 lag = 258 ms), or outside of it (T1-T2 lag = 688 ms). By contrast, the present masking experiment varied the proportion of seen trials quasi-continuously by manipulating the target-mask interval, thus allowing us to probe the transition to conscious access in a parametric manner. This design revealed that several ERP components such as P1b and N2, occurring before the late global P3, are already partially correlated with subjective reports, although their shape does not exactly match the nonlinearity of conscious access. Those observations suggest that the processing leading to conscious access for masked stimuli is not instantaneous, but follows a progressive dynamics of target-mask competition extending over a large duration (about 140–270 ms post-stimulus) and ultimately resulting in the P3.

One interpretation of these data could be that all components prior to the P3 reflect a progressive process of conscious access, whereas the P3 characterizes the final dichotomous result of this process (“seen” or “not seen”). This hypothesis, while clearly speculative, may reconcile the apparent all-or-none character of conscious reports [31] with the continuous and cumulative character of brain dynamics. As demonstrated in computer simulations of a global neuronal workspace [19,20], highly interconnected thalamo-cortical networks, although evolving continuously over time, may present a dynamical phase transition leading them, over a brief divergence period, into one of two radically distinct states (either global ignited or quickly decaying). Although the transition seems abrupt and its end result is sharply defined (essentially all or none), studies at a high temporal resolution, as performed here, reveal that the transition is characterized by a quick succession of transient intermediate states.

The localizations that we obtained from ERP source reconstructions suggest that the P3 relates to the activation of a highly distributed network with key nodes in inferior frontal, posterior parietal, and anterior temporal regions, as well as a joint amplification of activation in posterior occipito-temporal regions. Importantly, this late global activation occurs simultaneously in both hemispheres, regardless of the initial hemifield of target presentation. This observation fits with the hypothesis that conscious access is associated with a breakdown of local “modular” processing and the broadcasting of accessed information to many bilateral cortical regions through long-distance cortico-cortical connections including those of the corpus callosum [17].

The inferred localizations must be taken cautiously, because they represent an indirect inference based on the choice of one out of many possible cortical activity patterns compatible with scalp recordings. Thus, it will be important to cross-validate the present findings with anatomically more accurate methods. In that respect, it is noteworthy that our cortical source modeling solutions are highly compatible with intracranial recordings which indicate a distributed pattern of sources of the scalp P3 and, crucially, its bilateral origin irrespective of the stimulated hemifield [52]. With fMRI, Haynes, Driver, and Rees [29] used a parametric method similar to ours in order to search for brain activity patterns that covaried with stimulus visibility (which, in their case, traced a U-shaped curve). They found a correlation of conscious reports with activity in extrastriate visual cortex and in distant fronto-parietal regions. A similar fronto-parietal engagement during conscious access to visual stimuli has been described in several other fMRI studies [18,27,53–55]. At the single-cell level, neurophysiological recordings in the macaque monkey during a threshold tactile detection task have also reported a progressive increase in the correlation of neural activity with perceptual judgments [56] and a tight correlation of trial-by-trial subjective reports with late frontal activity [57].

### Theoretical Implications

In the present backward masking paradigm, conscious access was associated with the late activation of a broadly distributed cortical network, starting at a latency of ∼270 ms. Those findings are incompatible with models postulating that local amplitude modulations—confined to early striate, extrastriate, or occipito-temporal cortices—constitute the necessary and sufficient conditions for consciousness [7,11,12]. We do not dispute the fact that early extrastriate differences between masked and unmasked stimuli can be found, both in event-related potentials [7,17,58,59] and in fMRI [7,12,13,17,60,61]. Indeed, the present results confirm that early posterior components such as P1b and N2 are modulated by masking. However, by measuring visibility on a trial-by-trial basis over an entire range of SOAs, we found that these early ERPs (1) can occur without conscious perception, (2) do not exhibit the signature sigmoidal shape of subjective reports, and (3) do not differ for seen and not-seen targets. Note that these conclusions might have been missed, and we might have concluded erroneously that these early components correlate with conscious perception, if we had only compared two extreme states (heavily masked versus lightly masked), as was done in many studies. We therefore suggest that in brain-imaging studies of masking and conscious access, it is essential to manipulate masking strength quasi-continuously and to base inferences on the entire activation profile [29,51].

Lamme and colleagues [8–10] have proposed that conscious perception is not associated with the first feedforward pass of activation in visual cortex, but with a later feedback reverberation. They hypothesize that “localized recurrent processing” [10] associated with short-range interactions among posterior occipito-temporal brain systems is the primary mechanism of perceptual awareness. Our results are only partially compatible with this notion. We did observe a progressive build-up of activation in posterior areas as the strength of masking decreased, as indexed by the P1b and N2. Our source reconstructions suggest that the ipsilateral P1b, which correlated with subjective report, reflects a reverberation of visual activity in bilateral extrastriate cortices, while the later N2 component may be associated with recurrent processing in a broader though nonglobal occipito-temporo-parietal network. These results are therefore compatible with a reverberation of activation within an increasingly global network that starts within posterior visual areas [8,10], However, our results also indicate that, while those early components may contribute to the subsequent transition toward conscious access or to its failure, they do not yet correspond to a full-blown conscious state.

Our observations are most consistent with the theory of a global workspace formed by multiple distant associative areas including prefrontal, parietal, and temporal cortices [16,19,20]. In Figure 1, we outlined the predictions derived from this model concerning the effect of decreasing masking strength. Those main predictions were: (1) initial visual activation unaffected by masking; (2) progressive increase in activation depth as masking strength decreases; (3) non-linear divergence at a late stage; and (4) global reverberation simultaneously engaging a distributed set of prefrontal, parietal, and posterior occipito-temporal regions. All of these predictions were supported by the data. Most importantly, cortical source modeling clearly indicated a strong contribution of inferior frontal and posterior parietal cortices to the observed correlate of conscious access. This is the single observation that most clearly supports the proposed global workspace model relative to Lamme et al.'s proposal of “localized recurrent processing.”

We conclude with a brief consideration of the limits of the present work. First, our conclusion rests on the absence of early differences in brain activity correlating with subjective reports. We therefore cannot exclude that a more sensitive method such as single-cell recording or phase synchrony analysis would reveal consciousness-related events prior to the present suggested onset time of 270 ms [58]. Second, we used multiple statistical comparisons to compare various ERP components and isolate the P3 as the sole correlate of subjective reports. Since the corresponding statistics were weakly significant (see Table 1), our conclusions may be affected by a type I error (i.e., the P3 effect may be a false positive). However, the P3 effect was found statistically significant by two independent analyses (difference between seen and not-seen trials at SOA = 50 ms and sigmoidal shape as a function of SOA). Furthermore, the effect was predicted by our model as well as by several previous studies [30,51]. For these reasons, a type I error seems unlikely, though not fully ruled out.

A third limitation is that we cannot yet ascertain whether the late ERP events that we have identified are all necessary for conscious perception—it remains possible that another paradigm, perhaps side-stepping the need for overt report, or using only an objective criterion for perceptual awareness, would reveal that conscious perception can occur without such global activity or with only a subset of the distributed cortical regions observed here [10,62]. Fourth, correlation is not causation. We only report a correlation between subjective perception and late ERPs, but another method capable of interfering with brain activity patterns, such as transcranial magnetic stimulation [63], would be needed to establish whether these late events play a causal role in the conscious state. Finally, the global neuronal workspace model, while providing a good fit to the observed dynamics of conscious access, remains underspecified at both anatomical and functional levels. In the future, it will be essential to provide a more precise specification of the brain areas associated with conscious-level processing and their respective computational roles. The present research only begins to narrow down the search for the mechanisms of conscious access.

## Materials and Methods

### 

#### Participants.

Twelve right-handed native French speakers took part in the experiment (7 women and 5 men; mean age: 23 y, ranging from 21 to 29 y). All had normal or corrected-to-normal vision. Due to a hardware problem, the objective performance data from one subject were lost.

#### Design and procedure.

The stimuli (the numerals 1, 4, 6, or 9) were presented on a black background at the center of the computer screen using E-Prime software. Presentation times of the stimuli were synchronized with the vertical refreshing rate of the screen (60 Hz) and were systematically verified. The trial began when four peripheral moving dots converged to a central cross that the subject had to fixate constantly. Then, the target stimulus was presented for 16 ms at a random position among the four dots, forming a virtual square around the fixation cross (1.4 degrees above or below and 1.4 degrees right or left of the fixation cross). After a variable delay (SOA) between the onset of the prime and the onset of the target, a mask appeared at the target location (mask duration 250 ms). The mask was composed of four letters (two horizontally aligned M's and two vertically aligned E's) surrounding the target stimulus without superimposing or touching it. Six conditions of SOAs were randomly intermixed across trials (16, 33, 50, 66, 83, and 100 ms). In the mask-only condition, the target number was replaced by a blank screen with the same duration of 16 ms. After a subsequent delay of 800 ms (during which ERPs could be recorded without interference), the subject performed a forced-choice task of comparing the target numeral to the numeral 5, thus providing an objective measure of stimulus perception. Then, the subject evaluated the subjective visibility of the target on a continuous scale, in a similar way to the Sergent and Dehaene study of attentional blink [30]. The scale was materialized by a horizontal bar (26° × 2°), presented at the center of the screen, labeled “not seen” at left and “maximal visibility” at right, which participants were instructed to use in order to rate the visibility of the prime as finely and coherently as possible, without time pressure. Subjects moved a cursor on the scale by pressing two designated keys on the answer box, and then validated their choice by pressing a third key. The cursor, a vertical rectangle (1.2° × 2°), could take 21 contiguous positions on the scale, thus defining quasi-continuous steps of subjective visibility which were linearly converted into the range 0–100 (0 = not seen, 100 = full visibility). The initial cursor position was random. Previous results have established the reliability of this procedure to measure small changes in subjective perception. [2,30,31].

The experiment began with an explanation of the task and a visualization of the displays sequence. After a short training, the subject performed 592 randomly presented trials belonging to six different SOA conditions (64 trials for 16, 33, 83, and 100 ms; 128 for intermediate SOAs 50 and 66 ms, where pilot work led us to anticipate mixed seen and not-seen responses), and one “mask only” condition with no target (80 trials).

#### Behavioral data analysis.

For the objective measure, we calculated for each SOA and for each participant the performance in comparing the target with the numeral 5. For the subjective measure, we categorized the visibility ratings from the six conditions of SOA and the mask-only condition. When the target was absent, subjects hardly ever gave subjective ratings exceeding 25% of the scale (0.3% of trials). Conversely, at the longest SOA where the target was most visible, the ratings almost always exceeded 25% of the scale (97.4% of trials). At intermediate SOAs, the distribution of cursor positions reached a minimum around 25%, separating the data into two sets, one peaking at visibility zero (not-seen trials), and the other at maximal visibility (seen trials), replicating our earlier observations [2,30,31]. Thus, the value of 25% visibility was used as a cut-off between seen and not-seen trials. We then calculated for each SOA and each subject the percentage of seen trials (Figure 2C).

We used nonlinear regression to fit the curves obtained by a sigmoid defined as 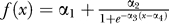 , where the α*_i_* are free parameters. The threshold was defined as the SOA for which the sigmoid curve reached its inflexion point, i.e., parameter α_4_. We studied the correlation of both measures of the conscious access threshold by performing linear regression across subjects between subjective and objective α_4_ parameters.

To measure if objective performance was better than chance or not, we performed for each SOA a *t*-test across subjects in comparison with chance level (performance equal to 50%). A single outlier subject was excluded. This subject showed a normal subjective threshold (52 ms), but high performance in the objective task with short SOAs (e.g., 82.8% correct at SOA = 33 ms), resulting in a low objective threshold (15 ms). Finally, two main analyses of variance (ANOVA) were performed respectively on the rate of “seen trials” and on the objective performance, with a single factor of SOA.

#### ERP methods.

ERPs were sampled at 250 Hz with a 128-electrode geodesic sensor net referenced to the vertex. We rejected voltage exceeding ±200 μV, transients exceeding ±100 μV, or electro-oculogram activity exceeding ±70 μV. The remaining trials were averaged in synchrony with mask onset, digitally transformed to an average reference, band-pass filtered (0.5–20 Hz), and corrected for baseline over a 250 ms window during fixation at the beginning of the trial.

#### ERP analysis.

*Separation of activation evoked by the mask and by the target:* Three consecutive stages were followed. (1) Alignment of all ERPs on mask onset. (2) Subtraction of ERP evoked by the mask alone from each of the other target + mask conditions. (3) Realignment of those subtracted data on target onset. This procedure is illustrated in Figure 3.

Different ERP waveforms were identified according to the positive or negative sign of the electric wave, its scalp topography, and the latency of its maximal peak according to stimulus onset. An additional criterion differentiated contralateral and ipsilateral waveforms according to the hemifield of stimulus presentation (right or left).

For each component and for each hemifield of stimulus presentation, we calculated its maximal (for positive waveforms) or minimal (for negative waveforms) amplitude and the corresponding latency within a predefined time window. Mean amplitude was computed over groups of electrodes representative of the topography of each scalp component: a group of parieto-temporal electrodes for P1a , P1b ,N1, and mask-evoked N1 (left electrodes: 52, 53, 59, 60, 61, 66, and 67; right electrodes: 78, 79, 85, 86, 87, 92, and 93), a group of occipito-temporal electrodes for N2 (left electrodes: 57, 58, 63, 64, 65, 69, and 70; right electrodes: 90, 91, 95, 96, 97, 100, and 101), and a group of central electrodes for P3 (7, 32, 55, 81, 107, and 129). The peak latency was calculated either relative to target onset or to mask onset, and the significance of a linear regression of these onsets with SOA was used to separate mask-evoked and target evoked components.

For the N2 wavefom, we also calculated onset and offset times. The onset time was defined as the latency at which the amplitude reached a threshold amplitude defined as half of the minimal waveform amplitude observed for SOA = 16 ms, and the offset time as the latency for which the amplitude fell back down to this threshold level. Again, these latencies were calculated either relative to target onset or to mask onset and we computed a linear regression with SOA of these latencies, in order to measure if the onset and the offset of these component were related to mask or target onset.

*Correlation of ERP components with subjective reports:* On mask-subtracted data and for the different conditions of SOA, a mean amplitude was calculated for each waveform component on a time window and for a group of electrodes precisely defined for each waveform. We thus obtained for each subject and for the different waveforms identified a vector of amplitude as a function of SOA. We performed an ANOVA on these amplitudes with factors of SOA and side of stimulus presentation (left or right), and also Students *t*-tests with the appropriated contrasts in order to test respectively the linear trend and the nonlinear sigmoidal increase of amplitude with SOA (see Results). Finally, we studied the correlation of these amplitudes with the subjective report of each subject using linear regression.

*Difference between seen and not-seen trials:* We subdivided the trials at SOA 50 ms between seen and not-seen trials according to the previous cut off defined as 25% of the subjective scale. Three subjects had to be excluded from this analysis because they did not have enough observations in one of the two categories. For the remaining nine subjects, a *t*-test comparing those two trial types was performed separately for each of the previously identified ERP components, and also a *t*-test contrasting target-present, not-seen trials with mask-only trials.

*Source modeling:* Cortical current density mapping was obtained using a distributed model consisting of 10,000 current dipoles. Dipole locations and orientations were constrained to the cortical mantle of a generic brain model built from the standard brain of the Montreal Neurological Institute, and warped to the standard geometry of the electroencephalogram (EEG) sensor net using the BrainSuite software package (http://brainsuite.usc.edu/). The warping procedure and all subsequent source analysis and surface visualization were processed with the BrainStorm software package (http://neuroimage.usc.edu/brainstorm). EEG forward modeling was computed with an extension of the overlapping-spheres analytical model [64]. Cortical current maps were computed from the EEG time series using a linear inverse estimator (weighted minimum-norm current estimate; see [65] for a review).

## Supporting Information

Figure S1 Reconstructed Cortical Activity in Different Regions of Interests in Response to Controlateral and Ipsilateral Targets The temporal evolution of cortical activity corresponding to mask-subtracted ERPs is shown separately for controlateral and ipsilateral targets and for the six conditions of SOA, in eight regions of interest. Activations are expressed in current density units (A.m). For reference, vertical lines show the peak latency of the observed target-evoked ERP components (P1a, P1b, N1, N2, and P3), and oblique lines show the latency range expected for mask-evoked components (mN1 and mN2). A precise sequence of cortical events is associated with the components observed on the scalp surface. (A–E) The fast temporal progression of activation across both occipito-parietal and occipito-temporal pathways within the first 250 ms. (D1 and D2) The asymmetry of this early activation, particularly for short SOAs (green curve, SOA = 33 ms) where activation remains largely contralateral to stimulus presentation is shown. (F–H) The late (>270 ms) sudden divergence of activation as a function of SOA, which occurs simultaneously in multiple bilateral areas of the frontal lobe. (A–E) The concomitant reactivation of bilateral parietal, temporal and. to a lesser extent. occipital cortices. The absent or much reduced difference between controlateral and ipsilateral sites in this late time period suggests a global broadcasting of conscious information.
(176 KB PPT)

Figure S2 Converging Evidence for a Late Fronto-Temporo-Parietal Correlate of Conscious Reportability The top panel shows the reconstructed cortical sources of the difference in evoked activity between a long SOA (100 ms), for which most targets were consciously seen, and a short SOA (33 ms), for which most targets were not seen. Similarly, the bottom panel shows the cortical sources of the difference between seen and not-seen trials at SOA 50 ms. In both cases, left, ventral, and right views are shown at *t* = 341 ms after target onset. Insets further show the average absolute current density in four bilateral frontopolar and anterior temporal regions of interest, as a function of time.
These two independent criteria for a neural correlate of conscious perception both point to the late activation of a distributed network including frontal, temporal and parietal areas. In particular, the reconstructed sources for seen and not-seen trials shows a first wave of activity (<250 ms) without any difference between seen and not-seen trials, followed by a later time window (270–400 ms), where seen trials elicit greater activity and which coincides precisely with the period where a nonlinear sigmoidal SOA effect is seen. Note, however, that the seen versus not-seen network also includes an additional broad activation increase along the ventral occipito-temporal pathway.
(627 KB PPT)

Figure S3 Evolution of ERPs in the SOA = 33 ms over a Group of Posterior Electrodes Contralateral to the Stimulus The blue curve was obtained by selectively averaging only over trials subjectively rated as “not seen,” whereas the green curve corresponds to the data reported in the main text and obtained by pooling across seen and not-seen trials. No statistical difference in amplitude was observed for any of the ERP components (P1a, P1b, N1, N2, P3, mN1, and mN2). In particular, the early components P1a and N1 were unchanged (*p* = 0.814 and *p* = 0.55, respectively) and remained statistically different from zero even when excluding the “seen” trials (*p* < 0.01 and *p* = 0.027). Thus, these components are essentially intact on not-seen masked trials, confirming earlier reports of preservation of these components independently of the subjects' conscious reports [30,35–37].
(38 KB PPT)

## Abbreviations

df: degrees of freedom
ERP: event-related potential
fMRI: functional MRI
SOA: stimulus onset asynchrony

## Note Added in Proof

Reference 66 was added during the proof stage and so is cited out of order in the text.

## References

[pbio-0050260-b001] Breitmeyer B (2006). Visual masking: Time slices through conscious and unconscious vision.

[pbio-0050260-b002] Del Cul A, Dehaene S, Leboyer M (2006). Preserved subliminal processing and impaired conscious access in schizophrenia. Arch Gen Psychiatry.

[pbio-0050260-b003] Kiefer M (2002). The N400 is modulated by unconsciously perceived masked words: Further evidence for an automatic spreading activation account of N400 priming effects. Brain Res Cogn Brain Res.

[pbio-0050260-b004] Naccache L, Gaillard R, Adam C, Hasboun D, Clémenceau S (2005). A direct intracranial record of emotions evoked by subliminal words. Proc Natl Acad Sci U S A.

[pbio-0050260-b005] Dehaene S, Naccache L, Le Clec'H G, Koechlin E, Mueller M (1998). Imaging unconscious semantic priming. Nature.

[pbio-0050260-b006] Haynes JD, Rees G (2005). Predicting the orientation of invisible stimuli from activity in human primary visual cortex. Nat Neurosci.

[pbio-0050260-b007] Pins D, Ffytche D (2003). The neural correlates of conscious vision. Cereb Cortex.

[pbio-0050260-b008] Super H, Spekreijse H, Lamme VA (2001). Two distinct modes of sensory processing observed in monkey primary visual cortex (V1). Nat Neurosci.

[pbio-0050260-b009] Lamme VA, Zipser K, Spekreijse H (2002). Masking interrupts figure-ground signals in V1. J Cogn Neurosci.

[pbio-0050260-b010] Lamme VA (2006). Towards a true neural stance on consciousness. Trends Cogn Sci.

[pbio-0050260-b011] Zeki S (2003). The disunity of consciousness. Trends Cogn Sci.

[pbio-0050260-b012] Tse PU, Martinez-Conde S, Schlegel AA, Macknik SL (2005). Visibility, visual awareness, and visual masking of simple unattended targets are confined to areas in the occipital cortex beyond human V1/V2. Proc Natl Acad Sci U S A.

[pbio-0050260-b013] Tong F (2003). Primary visual cortex and visual awareness. Nat Rev Neurosci.

[pbio-0050260-b014] Allison T, Puce A, Spencer DD, McCarthy G (1999). Electrophysiological studies of human face perception. I: Potentials generated in occipitotemporal cortex by face and non-face stimuli. Cereb Cortex.

[pbio-0050260-b015] Baars BJ (1989). A cognitive theory of consciousness.

[pbio-0050260-b016] Dehaene S, Kerszberg M, Changeux JP (1998). A neuronal model of a global workspace in effortful cognitive tasks. Proc Natl Acad Sci U S A.

[pbio-0050260-b017] Dehaene S, Naccache L (2001). Towards a cognitive neuroscience of consciousness: Basic evidence and a workspace framework. Cognition.

[pbio-0050260-b018] Dehaene S, Naccache L, Cohen L, Bihan DL, Mangin JF (2001). Cerebral mechanisms of word masking and unconscious repetition priming. Nat Neurosci.

[pbio-0050260-b019] Dehaene S, Sergent C, Changeux JP (2003). A neuronal network model linking subjective reports and objective physiological data during conscious perception. Proc Natl Acad Sci U S A.

[pbio-0050260-b020] Dehaene S, Changeux JP (2005). Ongoing spontaneous activity controls access to consciousness: A neuronal model for inattentional blindness. PLoS Biol.

[pbio-0050260-b021] Dehaene S, Changeux JP, Naccache L, Sackur J, Sergent C (2006). Conscious, preconscious, and subliminal processing: A testable taxonomy. Trends Cogn Sci.

[pbio-0050260-b022] Rees G, Kreiman G, Koch C (2002). Neural correlates of consciousness in humans. Nat Rev Neurosci.

[pbio-0050260-b023] Vuilleumier P, Sagiv N, Hazeltine E, Poldrack RA, Swick D (2001). Neural fate of seen and unseen faces in visuospatial neglect: a combined event-related functional MRI and event-related potential study. Proc Natl Acad Sci U S A.

[pbio-0050260-b024] Beck DM, Rees G, Frith CD, Lavie N (2001). Neural correlates of change detection and change blindness. Nature Neuroscience.

[pbio-0050260-b025] Marois R, Yi DJ, Chun MM (2004). The neural fate of consciously perceived and missed events in the attentional blink. Neuron.

[pbio-0050260-b026] Lumer ED, Rees G (1999). Covariation of activity in visual and prefrontal cortex associated with subjective visual perception. Proc Natl Acad Sci U S A.

[pbio-0050260-b027] Lau HC, Passingham RE (2006). Relative blindsight in normal observers and the neural correlate of visual consciousness. Proc Natl Acad Sci U S A.

[pbio-0050260-b028] Tononi G (2004). An information integration theory of consciousness. BMC Neurosci.

[pbio-0050260-b029] Haynes JD, Driver J, Rees G (2005). Visibility reflects dynamic changes of effective connectivity between V1 and fusiform cortex. Neuron.

[pbio-0050260-b030] Sergent C, Baillet S, Dehaene S (2005). Timing of the brain events underlying access to consciousness during the attentional blink. Nat Neurosci.

[pbio-0050260-b031] Sergent C, Dehaene S (2004). Is consciousness a gradual phenomenon? Evidence for an all-or-none bifurcation during the attentional blink. Psychol Sci.

[pbio-0050260-b032] Naccache L, Dehaene S (2001). The priming method: Imaging unconscious repetition priming reveals an abstract representation of number in the parietal lobes. Cereb Cortex.

[pbio-0050260-b033] Kunde W, Kiesel A, Hoffmann J (2003). Conscious control over the content of unconscious cognition. Cognition.

[pbio-0050260-b034] Merikle PM, Reingold EM (1990). Recognition and lexical decision without detection: unconscious perception?. J Exp Psychol Hum Percept Perform.

[pbio-0050260-b035] Vaughan HG, Silverstein L (1968). Metacontrast and evoked potentials: A reappraisal. Science.

[pbio-0050260-b036] Woodman GF, Luck SJ (2003). Dissociations among attention, perception, and awareness during object-substitution masking. Psychol Sci.

[pbio-0050260-b037] Kranczioch C, Debener S, Schwarzbach J, Goebel R, Engel AK (2005). Neural correlates of conscious perception in the attentional blink. Neuroimage.

[pbio-0050260-b038] Vorberg D, Mattler U, Heinecke A, Schmidt T, Schwarzbach J (2003). Different time courses for visual perception and action priming. Proc Natl Acad Sci U S A.

[pbio-0050260-b039] Ferrand L, Grainger J (1994). Effects of orthography are independent of phonology in masked form priming. Q J Exp Psychol [A].

[pbio-0050260-b040] Bridgeman B (1975). Correlates of metacontrast in single cells of the cat visual system. Vision Res.

[pbio-0050260-b041] Macknik SL, Livingstone MS (1998). Neuronal correlates of visibility and invisibility in the primate visual system. Nat Neurosci.

[pbio-0050260-b042] Kovacs G, Vogels R, Orban GA (1995). Cortical correlate of pattern backward masking. Proc Natl Acad Sci U S A.

[pbio-0050260-b043] Rolls ET, Tovee MJ, Panzeri S (1999). The neurophysiology of backward visual masking: Information analysis. J Cogn Neurosci.

[pbio-0050260-b044] Green MF, Glahn D, Engel SA, Nuechterlein KH, Sabb F (2005). Regional brain activity associated with visual backward masking. J Cogn Neurosci.

[pbio-0050260-b045] Thompson KG, Schall JD (1999). The detection of visual signals by macaque frontal eye field during masking. Nat Neurosci.

[pbio-0050260-b046] Nieuwenhuis S, Ridderinkhof KR, Blom J, Band GP, Kok A (2001). Error-related brain potentials are differentially related to awareness of response errors: evidence from an antisaccade task. Psychophysiology.

[pbio-0050260-b047] Brazdil M, Rektor I, Daniel P, Dufek M, Jurak P (2001). Intracerebral event-related potentials to subthreshold target stimuli. Clin Neurophysiol.

[pbio-0050260-b048] Brazdil M, Rektor I, Dufek M, Jurak P, Daniel P (1998). Effect of subthreshold target stimuli on event-related potentials. Electroencephalogr Clin Neurophysiol.

[pbio-0050260-b049] Keysers C, Perrett DI (2002). Visual masking and RSVP reveal neural competition. Trends Cogn Sci.

[pbio-0050260-b050] Keysers C, Xiao DK, Foldiak P, Perrett DI (2001). The speed of sight. J Cogn Neurosci.

[pbio-0050260-b051] Van Aalderen-Smeets SI, Oostenveld R, Schwarzbach J (2006). Investigating neurophysiological correlates of masking with magnetoencephalography. Adv Cogn Psych.

[pbio-0050260-b052] Clarke JM, Halgren E, Chauvel P (1999). Intracranial ERPs in humans during a lateralized visual oddball task: II. Temporal, parietal, and frontal recordings. Clin Neurophysiol.

[pbio-0050260-b053] Weidner R, Shah NJ, Fink GR (2006). The neural basis of perceptual hypothesis generation and testing. J Cogn Neurosci.

[pbio-0050260-b054] Kouider S, Dehaene S, Jobert A, Le Bihan D (2007). Cerebral bases of subliminal and supraliminal priming during reading. Cereb Cortex.

[pbio-0050260-b055] Kleinschmidt A, Buchel C, Hutton C, Friston KJ, Frackowiak RS (2002). The neural structures expressing perceptual hysteresis in visual letter recognition. Neuron.

[pbio-0050260-b056] de Lafuente V, Romo R (2006). Neural correlate of subjective sensory experience gradually builds up across cortical areas. Proc Natl Acad Sci U S A.

[pbio-0050260-b057] de Lafuente V, Romo R (2005). Neuronal correlates of subjective sensory experience. Nat Neurosci.

[pbio-0050260-b058] Melloni L, Molina C, Pena M, Torres D, Singer W (2007). Synchronization of neural activity across cortical areas correlates with conscious perception. J Neurosci.

[pbio-0050260-b059] Koivisto M, Revonsuo A, Lehtonen M (2006). Independence of visual awareness from the scope of attention: An electrophysiological study. Cereb Cortex.

[pbio-0050260-b060] Bar M, Tootell RBH, Schacter DL, Greve DN, Fischl B (2001). Cortical mechanisms specific to explicit visual object recognition. Neuron.

[pbio-0050260-b061] Grill-Spector K, Kushnir T, Hendler T, Malach R (2000). The dynamics of object-selective activation correlate with recognition performance in humans. Nat Neurosci.

[pbio-0050260-b062] Block NJ (1974). Fictionalism functionalism and factor analysis. Proceedings of the Biennial Meeting of the Philosophy of Science Association.

[pbio-0050260-b063] Beck DM, Muggleton N, Walsh V, Lavie N (2006). Right parietal cortex plays a critical role in change blindness. Cereb Cortex.

[pbio-0050260-b064] Huang MX, Mosher JC, Leahy RM (1999). A sensor-weighted overlapping-sphere head model and exhaustive head model comparison for MEG. Phys Med Biol.

[pbio-0050260-b065] Baillet S, Mosher JC, Leahy RM (2001). Electromagnetic brain mapping. IEEE Signal Processing Magazine.

[pbio-0050260-b066] Di Lollo V, Enns JT, Rensink RA (2000). Competition for conciousness among visual events: the psychophysics of reentrant visual processes. J Exp Psychol Gen.

